# Ozonated autohemotherapy: protection of kidneys from ischemia in rats subjected to unilateral nephrectomy

**DOI:** 10.1186/1471-2369-12-61

**Published:** 2011-11-14

**Authors:** Chiara Foglieni, Alessandro Fulgenzi, Daniela Belloni, Clara Sciorati, Elisabetta Ferrero, Maria Elena Ferrero

**Affiliations:** 1 Clinical Cardiovascular Biology Laboratory, San Raffaele Scientific Institute, Milano, Italy; 2 Dipartimento di Morfologia Umana e Scienze Biomediche - Città Studi, Università degli Studi di Milano, Milan, Italy; 3 Department of Oncology, IRCCS H San Raffaele, Milan, Italy; 4 Laboratory of Lymphoid Malignancies, San Raffaele Scientific Institute, Milan, Italy

## Abstract

**Background:**

Ozonated autohemotherapy (OA) has been previously successfully used in the treatment of patients affected by peripheral occlusive arterial disease. OA consists of an intrafemoral reinfusion of autologous blood previously exposed to a mixture of oxygen/ozone (O_2_/O_3_). This study analyzes the effects of OA in protecting rat kidney from ischemia and ischemia/reperfusion damage.

**Methods:**

We performed OA 30 min before the induction of 60 min renal ischemia or at the induction of 60 min postischemic reperfusion in rats subjected to unilateral nephrectomy. In addition, to evidence the possible protection induced by O_2_/O_3 _on endothelial functions, the present study analyzes the in vitro effects of O_2_/O_3 _on oxygen consumption by human umbilical vein endothelial cells (HUVEC).

**Results:**

1) OA preserves rat kidney functions and architecture, as demonstrated by the improved levels of serum creatinine and blood urea nitrogen and by histology; 2) such protection does not correlate with the increase of plasmatic nitric oxide, but is compatible with a focal renal increase of renal βNADPH-diaphorase; 3) treatment of HUVEC with O_2_/O_3 _significantly increases both the rate of oxygen consumption and the mitochondrial activity assessed by confocal microscopy.

**Conclusion:**

The preservation of the mitochondrial activity of endothelium could in vivo limit the endothelial dysfunction provoked by the Isc or Isc/R processes.

## Background

The controversial debate on the beneficial versus the toxicological actions of ozone (O_3_), is still open [1]. However, if O_3 _is judiciously used within the precisely determined therapeutic window, it does not cause toxic effects [2-4]. Few relevant clinical applications demonstrate that Oxygen-Ozone (O_2_/O_3_) autohemotherapy (OA, i.e. slow re-infusion of autologous blood previously exposed to a O_2_/O_3 _mixture), is useful in cardiovascular disorders and tissue ischemia, as well as supportive in viral infections through stimulation of the immune system [1,2]. In patients with peripheral occlusive arterial disease, OA improves hemorheological parameters and O_2 _delivery to tissues. [3]. Moreover, OA improves the capability of erythrocytes to deliver O_2 _to ischemic tissues, and finally induces a localized release of protective nitric oxide (NO), CO and growth factors from platelets [1,2].

Ischemia (Isc) and Ischemia/Reperfusion (Isc/R)-related injuries are major causes of acute kidney injury following renal transplantation. Despite tolerance induction, long-term surviving kidney allografts can develop chronic damages [4,5]. Among the O_2_-based methods, O_2_/O_3 _preconditioning, i.e. intra-peritoneum injection of O_2_/O_3 _mixture before ischemic damage, is an efficient protective system from rat Isc/R in both liver [6] and kidney [7,8]. In the renal model, O_2_/O_3 _preconditioning increases the expression of endothelial and inducible forms of NO synthase (eNOS, iNOS, respectively), and the NO release [9], with a mechanism similar to that induced by Isc preconditioning. The latter is an experimental model of brief, sequential ischemic events, that results in protecting against subsequent ischemic episodes. In comparison with both Isc and O_2_/O_3 _preconditioning, OA could have the considerable advantage of being feasible in humans. In our knowledge, no clinical or experimental data are presently available on OA role in protecting from renal Isc/R injury. Aim of the present study was to investigate if OA could be proposed to improve renal damage following Isc and Isc/R, in comparison with O_2 _alone.

Endothelial cells have been proposed to be elective targets of the positive molecular effects of ozone and its derived species formed during blood ozonation [10]. As mechanism of action, we have hypothesized that O_2_/O_3 _could locally increase the O_2 _availability for endothelial cells, supporting their resistance to dysfunction, thus contributing to protection of organs from Isc/R damage. Indeed, we evaluated: the effects of OA on renal morphology and function in rats subjected to Isc or Isc/R; plasmatic nitric oxide (NO) levels and renal iNOS isoform expression, because of the cytoprotective role of NO in Isc/R [11,12]. The effects of O_2_/O_3 _on endothelium were also investigated on rat renal arteries through expression of CD31/PECAM1, and in vitro through measurements of O_2 _consumption, mitochondrial oxidative activity and cell metabolism.

## Methods

### Animals

Male adult Wistar rats (Harlan Italy, S.Pietro al Natisone, Udine, Italy), 230-250 g, were used. The rats had free access to standard pelleted food and water and were maintained at temperature of 22 ± 1°C with a 12 h light/dark cycle. All experimental procedures conformed to the "guide for the care and the use of Laboratory Animals published by the US National Institute of Health (NIH publication NO: 85-23, revised 1996), according to the animal welfare regulations of the Italian local authorities (Ethical Committee of the University of the Studies of Milan, Italy).

### Experimental groups

We submitted rats to Isc followed by brief R time, because kidney damages occur early during R and previous authors already studied the effects of ozone-oxidative preconditioning after long R time [7-9]. Moreover, we submitted rats to unilateral nephrectomy to avoid the controlateral kidney influence before to induce Isc.

The rats were randomly assigned to 8 groups (n = 7 rats/group). All the rats were treated with autologous blood groups 1 to 8 by intrafemoral injection. Groups 1 and 5 were unoperated (e.g. controls); groups 2 and 6 sham operated (e.g. rats undergoing the surgical procedure without clamp of the left renal artery); groups 3 and 7 were ischemized (Isc); groups 4 and 8 were ischemized and reperfused (Isc/R). In groups 2 to 4 and 6 to 8 the rats underwent right kidney nephrectomy before any other treatment. In groups 1, 2, 3, 4. autologous blood was treated with O_2 _alone. In groups 5, 6, 7, 8 autologous blood was treated with O_2_/O_3 _mixture (OA) Intrafemoral injection of autologous blood or OA was performed 30 min before the rat killing in groups 1 and 5, or 30 min after right kidney removal in groups 2 and 6, respectively. Groups 3 and 7 received a single intrafemoral injection of the blood 30 min before Isc (indeed underwent Isc). Groups 4 and 8 received a single intrafemoral injection of the blood at the time of clamp removal (e.g. at R). Sampling was performed after 60 min reperfusion. The characteristics of the different groups are reported in Table 1.

**Table 1:** Experimental groups of rats

GROUPS		O_2_/O_3_	AUTOHEMOTHERAPY
GROUP 1	CONTROLS	-	30' before killing

GROUP 2	"SHAM OPERATED"	-	30' after right kidney removal

GROUP 3	Isc	-	30' before Isc

GROUP 4	Isc/R	-	at R

GROUP 5	CONTROLS	+	30' before killing

GROUP 6	"SHAM OPERATED"	+	30' after right kidney removal

GROUP 7	Isc	+	30' before Isc

GROUP 8	Isc/R	+	at R

### Isc/R model

Rats were anesthetized inhaling a mixture of halothane 2% (Hoechst, Milano, Italy) in oxygen. They were placed on a temperature-regulated table (38°C) (Ugo Basile, Comerio, Lecco, Italy) to maintain body temperature. Isc was induced in kidneys by clamping the left renal artery and the left renal vein for 60 min with a microsurgical clamp. R was obtained by removing vascular clamp and lasted 60 min. During the surgical procedure heart rate and mean arterial blood pressure were monitored. At the end of Isc or of Isc/R, rats were exanguinated at the aorta bifurcation level, blood samples were recovered, and kidneys were collected from 4 animals/group and processed for histological analysis. During the surgical procedure the heart rate and the mean arterial blood pressure were monitored, as previously described [13].

### Ozonated autohemotherapy

First of all we set up the optimal O_2_/O_3 _concentration. An ozone generator (Multiossigen, Gorle, Bergamo, Italy) was used to erogate the O_2_/O_3 _gas mixture, composed of an equivalent volume of ozone-oxygen (1:1 volume relationship). Ozone concentration in the mixture was 50 μg/ml, per ml of blood, known to not cause oxidative injury *in vivo *[14]. Rat blood (1 ml) was drawn from the caudal vein into a sterile glass tube; and 20 μl heparin (5000 U.I./ml, Vister, Parke-Davis, Lainate, Milano, Italy) was used as an anticoagulant. Tube was connected to the ozone generator and 1 ml blood *ex vivo *exposed to 5 ml of gas mixture. During the exposure to the gas mixture (4 min) blood was continuously and gently shaken until it appeared light red, then was re-infused into the left femoral vein of the donor rat. No other medication was given. Autohemotherapy with the administration of medical oxygen was performed as a control treatment.

### Functional studies

Serum creatinine was measured using a modified Jaffe's reaction and blood urea nitrogen was measured on the AEROSET system (Abbott Laboratories, Abbott Park, IL) [13].

### Histopathology of rat kidneys

Collected kidneys were fixed and processed as previously described [15]. Renal damage was evaluated on 4 to 6 sections stained with Hematoxylin/Eosin as tubular epithelial cell necrosis, tubular dilation, protein casts and medullary congestion. The alterations were semi-quantitatively graded (- = absent, + = barely present, ++ = moderate, +++ = severe) [16] instead of being submitted to statistical analysis, which was scarcely reliable in our conditions. Additional 4 to 6 sections from O_2_/O_3_-treated rats were submitted to βNADPH diaphorase for evaluating NOS activity. In fact βNADPH diaphorase histochemistry reflects the expression of total NOS in the rat kidney tissue [17]. Slides were incubated with 1 mM βNADPH/0.2 mM nitroblue tetrazolium/100 mM Tris-HC1 buffer pH 8.0 containing 0.2% Triton X-100 for 60 min at 37°C as described [13]. A pathologist who worked blindly analyzed the sections using Eclipse 55i microscope equipped with a DS-L1 camera (Nikon, Tokyo, Japan).

### Nitrite/Nitrate determination

At the end of Isc and of Isc/R, 1 ml of heparinized (not coagulated) blood was drawn at the aorta bifurcation of each rat to measure the NO concentrations. Blood samples underwent centrifugation and plasma aliquots were stored at -21°C until determination. Plasma nitrite/nitrate were determined using the Greiss reaction that measures combined oxidation products of NO, plasma nitrite (NO_2_^-^) and nitrate (NO_3_^-^) after reduction with nitrate reductase in a colorimetric assay [18]. Standard curves with increasing concentrations of sodium nitrate and sodium nitrite were run in parallel.

### Isolation and culture of HUVEC

Human umbilical vein endothelial cells (HUVEC) were isolated from human cord by collagenase treatment as described [19] and cultured in 1% gelatin-coated flasks (Falcon; Becton Dickinson, Bedford, MA, USA) using endotoxin-free Medium 199 (BioWhittaker, Cambrex Bio Science Verviers, Belgium), containing 20% heat-inactivated fetal bovine serum (FBS, Hyclone, Logan, UT, USA), 1% bovine retinal-derived growth factor, 90 μg/ml heparin, 100 I.U./ml penicillin, and 100 μg/ml streptomycin (Biochrom, Berlin, Germany). All experiments were carried out with HUVEC at passage 1-4.

### O_2 _consumption by HUVEC

Consumption of O_2 _was measured using the Biological Oxygen Monitor System 5300A (YSI Incorporated, Yellow Springs, Ohio, USA) and Clark-type-polarographic O_2 _electrode. The data were recovered with WinWedge software (TAL Technologies, Philadelphia, PA, USA).

During O_2 _consumption measurements, the cells were placed in thermostatically-regulated chambers (37°C) equipped with a magnetically stirred circulator. The instrument calibration was performed considering the levels of O_2 _as 24% (= μl of O_2_/ml, corresponding to the volume of O_2 _dissolved in aqueous medium at 1 atm and 37°C) [20]. O_2 _consumption values were recorded every 5 sec for 15 to 60 min. After 5 min of acclimatization, O_2_/O_3 _mixture was added in about 10 seconds (2 ml, at the ozone concentration of 50 μg O_3_/ml) through a PE 50 polyethylene tube [21].

### Measure of ATP and LDH levels in HUVEC

Measure of oxygen consumption in HUVEC has been associated to the dosage of adenosine 5' triphosphate (ATP) and lactate dehydrogenase (LDH). Briefly, HUVEC seeded on 6 well and 24 well plates respectively, were exposed to the O_2_/O_3 _mixture (2 ml at the concentration of 1 μl/ml) for about 2 minutes; after that, ATP and LHD contents were measured at different time intervals (e.g. 5, 30, 60, 90, 120 min). The assays were performed in quadruplicate. ATP luminescence assay kit (Invitrogen, San Giuliano Milanese, Milano, Italy) and LDH assay kit (Cytotoxicity Colorimetric assay kit, Oxford Biochemical Research INC, Oxford, MI, USA) have been used. ATP levels have been referred to HUVEC protein content, whereas LDH to the volume (ml) of cell suspension.

### HUVEC protein content assay

Cell protein content has been measured by the colorimetric method, using Coomassie brilliant blue G-250 (BioRad Laboratories, Richmond, CA, USA). Cells were previously lysated as described in the manifacture protocol. Bovine serum albumin was used as standard (BioRad protein assay).

### Confocal microscopy on HUVEC

Mitochondria and nuclear activities were labeled on living HUVEC, grown on glass coverslips (10^5 ^cells/coverslip) with mitoTraker CMX-ROS (250 nM, 40 min, 37°C) (Molecular Probes, Eugene, OR) and with acridine orange (Sigma-Aldrich, St. Louis, Missouri) [17], prior to insufflate cells with 1 ml O_2_/O_3_. mixture.

Time-course confocal microscopy was performed using a Leica TCS SP2 AOBS (Leica Microsystems GmbH, Wetzlar Germany) confocal microscope. Free projection max (FPM) images were obtained from single channels-collected Z-series, acquired before the O_2_/O_3 _treatment, at the moment of O_2_/O_3 _addition (t 0), after 5, 10, 20, 30, 60, 120, 180 min (t 1-t 7) from O_2_/O_3 _addition. Single-channel FPM were merged with Adobe Photoshop CS.

### Statistical Analysis

Results were expressed as the mean ± SEM. We performed Student's t test to analyse the distribution of the groups. Indeed, we performed Kolmogorov-Smirnov test and ANOVA test to analyse the differences between groups; p values less than 0.05 were considered significant.

## Results

In the rats studied the heart rate did not significantly vary during the experimental procedure, whereas mean arterial pressure significantly increased during ischemia (data not shown) [13]. Post-surgery polyuria or oliguria were not evidenced.

### OA improves renal dysfunction induced by both Isc and Isc/R

**Figure 1:**
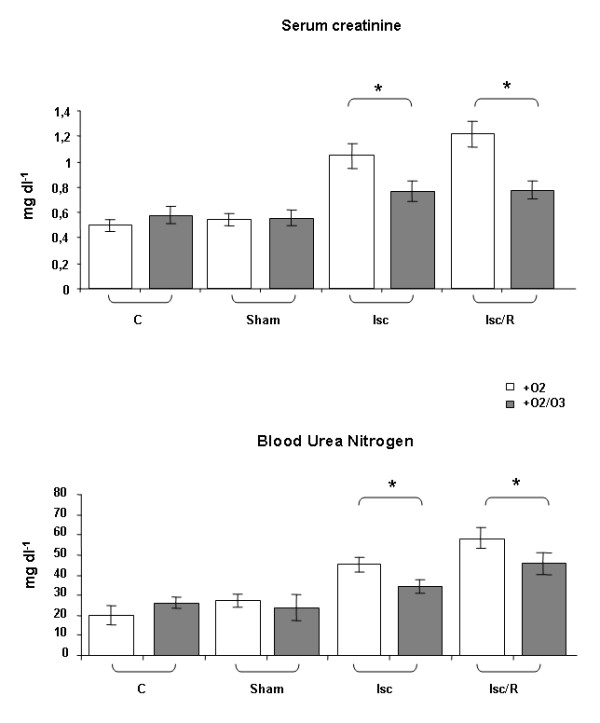
**Effect of ozonated autohemotherapy on renal function after Isc and Isc/R**. The rats received an intrafemoral injection of their blood driven from caudal vein and treated with O_2 _or a mixture of O_2_/O_3_; the injection was performed 30 min before Isc or at the induction of post-Isc R. Serum creatinine and blood urea nitrogen levels were measured. Isc and Isc/R rats subjected to O_2_/O_3 _autohemotherapy showed significant reductions of serum creatinine and blood urea nitrogen levels as compared with animals subjected to O_2 _autohemotherapy. Abbreviations: C = controls rats, Sham = sham-operated rats, Isc = rats submitted to 60 min of ischemia by clamping the renal artery; Isc/R = rats submitted to 60 min of ischemia followed by 60 min reperfusion. *p < 0.05

We compared renal functions of rats subjected to O_2 _and O_2_/O_3 _autohemotherapy (A vs. B, Table 1). Both Isc and Isc/R significantly increased serum creatinine and blood urea nitrogen levels in respect to controls and sham-operated animals. Notably, pre-treatment with OA (group B) protected kidneys from damage due to Isc or Isc/R, keeping serum creatinine and blood urea nitrogen at values comparable to those measured in controls and in sham-operated rats, but significantly lower than Isc- and Isc/R- induced animals, not submitted to OA (Figure 1).

### OA protects kidneys from histological damages inflicted by Isc and Isc/R

Tubular alteration is commonly considered an indicator of Isc renal damage, and alterations in intrarenal microcirculation and O_2 _handling can contribute to organ dysfunction, leading to Isc-related acute kidney injury [7,11]. Early alteration of peritubular capillary blood flow during reperfusion has been previously related to loss of normal endothelial cell function and Isc preconditioning prevents endothelial dysfunction [22,23].

**Figure 2:**
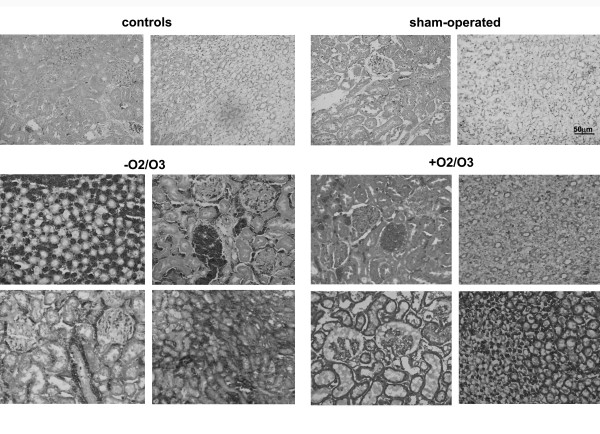
**Histological features of kidneys from rats submitted to O_2 _or toO_2_/O_3 _autohemotherapy**. Hematoxylin/Eosin stain of sections from control and sham operated rats show that surgical treatment did not affect normal renal morphology displayed by histologic findings (upper row images). Isc and Isc/R, in the presence of O_2 _therapy, dramatically altered the tubular and glomerular architectures and viability (middle and lower rows, left images). The O_2_/O_3 _autohemotherapy reduced the entity of alterations, especially the tubular damage and congestion of the medulla (middle and lower rows, right images) (original magnification x200).

Histological findings were summarized in Table 2 and Figure 2. Kidneys from Isc- and Isc/R- submitted rats displayed tubular necrosis, medullary congestion and glomerular damages in comparison to kidneys from control and sham-operated rats, accordingly with our previous findings [13]. In particular, focal glomerular damage, i.e. persistence of protein casts engulfing glomerular capillaries and Bowman spaces, was observed in Isc/R animals. OA treatment was able to reduce this tubulo-medullary damages, in particular preserved glomerular morphology.

**Table 2:** Histopathology of rat kidneys

	Group	Tubular necrosis	Tubular dilation	Protein casts	Medullary congestion	Glomerular damage	Interstitial stasis
	**1 **control	-	-	-	-	-	-
	
**A**	**2 **sham-operated	-	-	-	-	-	+/-
	
	**3 **Isc	-	+	+	+/++	-	++
	
	**4 **Isc/R	+	++	+++	+	-	+

	**5 **control	-	-	-	-	-	-
	
**B**	**6 **sham-operated	-	-	-	-	-	+/-
	
	**7 **Isc	+	-	+/-	+	+	++
	
	**8 **Isc/R	+	+	++	+/+/-	+	+

(A) Autologous blood + O_2_

(B) Autologous blood + O_2_/O_3_

### OA effects on: nitrite/nitrate, βNADPH-diaphorase, iNOS, CD31/PECAM1

**Figure 3:**
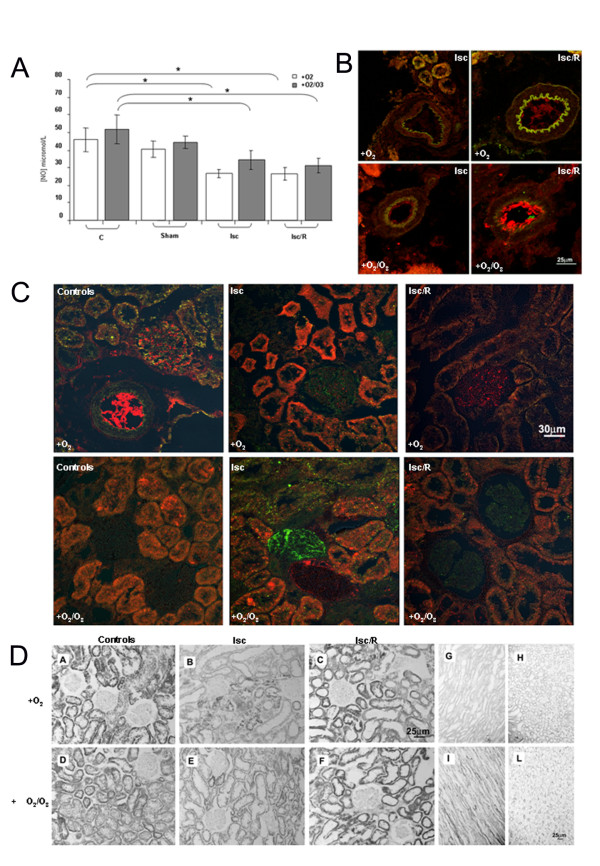
**Effect of O_2_/O_3 _therapy on plasmatic nitrite/nitrate and renal iNOS activity**. In panel A are reported plasmatic nitrite/nitrate levels; the mean values/group of animals obtained by Greiss colorimetric assay are expressed in micromoles/L. Rats subjected to Isc or Isc/R that received or not O_2_/O_3 _autohemotherapy showed reduced levels of NO as compared with corresponding controls C. Abbreviations: Sham = sham-operated rats. Isc = ischemia; Isc/R = ischemia/reperfusion. *p < 0.05. Panels B, C show iNOS (green) and CD31 (red) expression by confocal microscopy on representative sections from renal cortex of rats treated with OA (+O_2_/O_3_) or not. Renal arterioles (panel B) display no iNOS. CD31 is bright comparably to control (cfr.with panel C, upper left) on the arteriolar endothelial lining of Isc/R +O_2_/O_3 _rats only. Glomeruli display an increased iNOS in Isc animals, brighter in rats +O_2_/O_3 _than in the others. Very faint iNOS is focally observed in sections from the untreated controls and in Isc/R +O_2_/O_3_. In panel D the renal βNADPH diaphorase oxidative activity is shown as blue formazan signal on kidney sections. No difference in the renal cortex signal was seen between presence or absence of OA, but Isc animals display an inhomogeneous loss, whereas Isc/R an increase of the tubular signal respect to controls. Conversely medullary tubular cells of Isc and Isc/R rats submitted to OA show an higher βNADPH diaphorase signal than all the others.

As expected, both Isc and Isc/R significantly decreased plasmatic nitrite/nitrate levels. OA did not significantly affect these levels (Figure 3A). Endothelial damage was investigated locally, through the expression of the endothelial marker CD31/PECAM1 in renal tissue. Its bright expression was evident on endothelial cells of cortical arterioles (Figure 3B) from OA-treated Isc/R rats at a level comparable to control animals, and to a lesser extent in OA-treated Isc rats. In animals not submitted to OA, a faint signal was detected in Isc/R and no label in Isc. Overall, these results indicate an endothelial alteration when rats undergoing Isc or Isc/r did not receive OA. Consistently, the inducible isoform of NOS, iNOS (Figure 3C), appeared focally and weakly expressed on glomerular capillaries of Isc, but not Isc/R rats in the absence of OA. In OA-treated rats iNOS was weak in Isc/R, but intense in Isc on glomerular capillaries, suggesting an activation of iNOS by OA treatment. Some CD31/PECAM1 was focally observed into glomerular capillaries, mostly in controls animals.

The isoform non-specific NOS activity, evaluated by βNADPH-diaphorase histochemistry, which indicated βNADPH oxidation in the renal tissue (Figure 3D), showed a decrease of both glomerular and tubular signal in damaged areas of the cortex, and of tubular activity in the medulla of Isc animals as compared to controls. This focal loss was more evident in the absence than in the presence of OA. Of note, areas of βNADPH-diaphorase intense signal were observed in the less injured cortical regions of Isc rats renal cortex. In kidney of rats submitted to OA an increase with respect to controls of tubular signal was observed in the cortex and in the medulla.

### O_2_/O_3 _mixture improves O_2 _consumption of HUVEC via increase of mitochondrial activity

**Figure 4:**
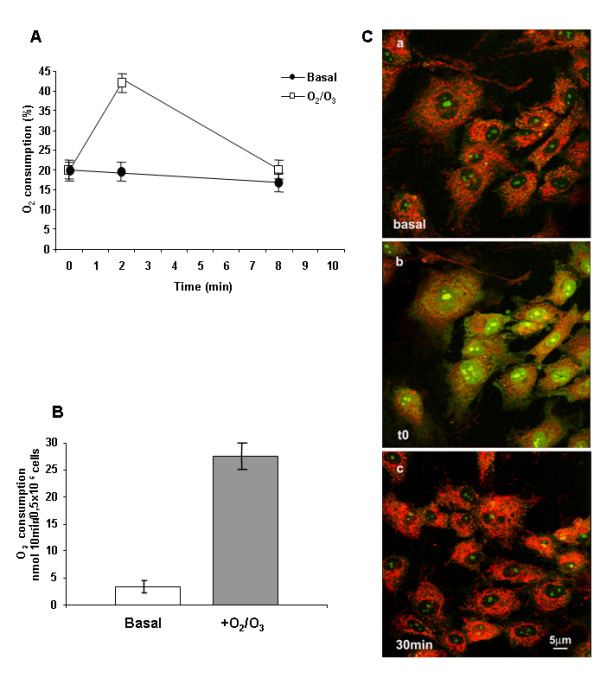
**Oxygen consumption and mitochondrial activity by HUVEC**. Panel A shows the O_2 _consumption of HUVEC measured by means of a Clark electrode. After a brief termic acclimatization, 2 ml of O_2_/O_3 _mixture containing 50 μgO_3_/ml was gently added to HUVEC medium (time 0). Two min after the addition of O_2_/O_3_, the availability of oxygen increased around twofold. The rate of oxygen consumption significantly increased with respect to basal conditions (cells untreated with O_2_/O_3 _mixture). The histogram in panel B compares total O_2 _consumed in 10 min in basal conditions and after the addition of the O_2_/O_3 _mixture, displaying a 6 times increase in O_2 _consumption for HUVEC treated with O_2_/O_3 _. Confocal microscope 2D pictures of HUVEC labeled with mito Traker CMX-ROS red and Acridine Orange green are represented in panel C. Image **b**, compared with image **a**, which shows the basal signal, displays an increase in both signals paralleling the infusion of O_2_/O_3 _at time 0. Image **c **demonstrates that the increase in mitochondrial respiration and in nuclear activity reverts to basal activities after 30 min from O_2_/O_3 _addition.

HUVEC in basal conditions displayed poor O_2 _consumption. Administration of O_2_/O_3 _mixture early increased O_2 _disposal, that was rapidly consumed (Figure 4A). The total amount of O_2 _consumed by O_2_/O_3_-treated HUVEC in 10 min, increased six-fold in comparison with untreated cells (Figure 4B). Confocal microscopy of HUVEC, 2 min after addition of the O_2_/O_3 _mixture (t0), showed a marked increase of both mitochondrial activity and Acridine orange signal as compared to basal fluorescence (Figure 4C panels a, b). Changes are compatible with the increased intracellular bioavailability of O_2 _suggested by the rapid rise in O_2 _consumption (Figure 4A). The effect reverted slowly to basal after 30 min from O_2_/O_3 _exposure (Figure 4C panel c). Confocal analysis performed 3h after O_2_/O_3 _exposure, demonstrated that HUVEC mitochondrial activity and Acridine orange signal were comparable to basal (data not shown), thus indicating the absence of metabolic degeneration following the treatment.

### OA increases ATP production, without affecting LDH, in HUVEC

**Figure 5:**
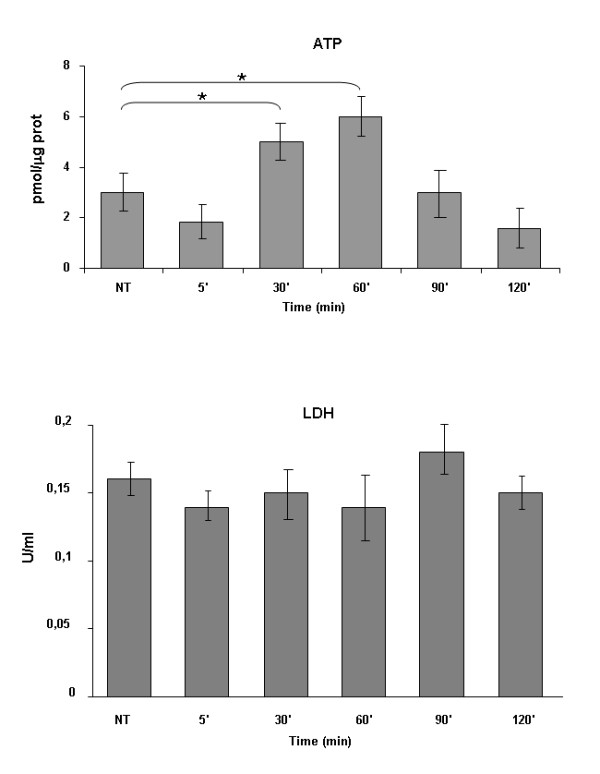
**ATP and LDH levels in HUVEC at different times from exposure to O_2_/O_3 _mixture**. The data represent the m ± SEM of 4 experiments. *P < 0.05 vs NT (not treated), e.g. not exposed to O_2_/O_3 _mixture.

After brief O_2_/O_3 _exposure, HUVEC were maintained in culture for up to 2 h. Total cellular ATP content, expressed in pmol/mg cellular protein, significantly increased at 30 and 60 min (Figure 5, upper panel) whereas LDH levels, expressed in U/ml, did not significantly vary (Figure 5, lower panel). ATP significantly increased 30 min after O_2_/O_3 _exposure, in agreement with the increase in mitochondrial activity (Figure 4C), suggesting that OA improves mitochondrial function.

## Discussion

The main objective of this study is to investigate if the OA, which is used in patients affected by PAOD, could be proposed as a possible method to reduce damage due to Isc and/or postischemic R. We tested our hypothesis in vivo using an experimental model of Isc and Isc/R in rat kidney and in vitro using the possible target of Isc damage, e.g. HUVEC. In the present work blood samples were exposed to a mixture of O_2_/O_3_, , and then re-infused in the donor rat, and compared to that exposed to O_2 _alone before re-infusion. The advantages of our proposed therapy over preconditioning is to reduce the number of treatments in the time and to permit a more direct availability of ozone to the Isc organ due to intravenous treatment. We raised the question whether the OA was effective in the protection against damage due to renal Isc/R, often provoked in patients by atherosclerotic renal artery stenosis.. Since the damage due to R is very early [23], we decided to use a short time of reperfusion (e.g. 1 h), also in consideration that some other previous works [7,9] have already reported the usefulness of O_2_/O_3 _treatment at longer reperfusion times. Our results show that OA significantly improves kidney functional parameters compromised by Isc and Isc/R. In fact, increases of serum creatinine and blood urea nitrogen levels are significantly reduced following OA (Figure 1). Histology shows that OA significantly reduces medullary congestion and tubular dilation provoked by Isc and Isc/R, and only slightly reduces protein casts (Figure 2 and Table 2). However, the protection on the renal function induced by OA does not correlate with an increase in NO production. An activation of iNOS by OA treatment is shown the alteration of NO/NOS pool occurring after Isc injury may play a protective or a damaging role. The analysis of the βNADPH diaphorase shows that the OA increases the enzyme activity in the cortical tubules and in the glomeruli of the kidney, only after Isc, suggesting an increase in NO local production following the blood supply interruption. In rat kidneys, increased glomerular βNADPH diaphorase, e.g. local synthesis of NO, after renal Isc, seems to be a protective mechanism that counteracts vasoconstrictor and inflammatory phenomena occurring during the R period. However, as previously reported, since Isc/R injury can activate inflammatory reactions, Isc/R-induced renal NO level may be related to a increase of nuclear factor kappa B-dependent pro-inflammatory factors, which play a major role in the activation of iNOS during the inflammatory process [24]. Moreover, increased NO in reperfused kidney might be greatly enhanced by a simultaneous increase in superoxide radicals [25]. In our experimental model OA seems to be protective against Isc/R injury via NO generation.

Endothelial injury occurs rapidly after an ischemic insult [23] and relates to reduced mitochondrial activity [15]. Because Krebs cycle and mitochondrial mass are early victims of endothelial dysfunction [26], to better evidence the possible influence of O_2_/O_3 _treatment on the modulation of endothelial dysfunction, we have determined the mitochondrial functions of HUVEC through their capability to consume O_2_. Recently, it has been demonstrated that decrease in O_2 _consumption by pulmonary artery endothelial cells from patients affected by idiopathic pulmonary arterial hypertension was related to a decreased mitochondrial activity for these cells [27]. Previously, the effect of ozonated serum on HUVEC has been investigated and a dose-dependent increase of hydrogen peroxide as the main mediator of ozone action (possibly through an increase of NO production) was shown [28]. In our experiments, HUVEC display a low rate of oxygen consumption in basal conditions. The addition of O_2_/O_3 _mixture significantly increases the oxygen availability and the rate of oxygen consumption (Figure 4A and 4B). In parallel, confocal microscopy evidences a marked increase of mitochondrial activity, as revealed by mito Traker (Figure 4C, red signal). We can argue that, whereas in basal conditions ATP is generated nearly equivalently by glycolysis and cellular respiration [27], O_2_/O_3 _treatment stimulates the mitochondrial energetic metabolism of HUVEC, as shown, within 30-60 min, by a significant increase in ATP levels. Because in the same time intervals LDH levels of HUVEC do not significantly vary, the increase of ATP is attributable to the increase of mitochondrial instead of glycolytic pathway. Indeed, the O_2_/O_3 _mixture could restore the energy production also *in vivo*, when the reduction of available O_2 _due to Isc impairs endothelial cell respiration, favouring only their glycolytic activity. ATP reverted to basal levels after 90 min exposure to O_2_/O_3 _mixture, indicating that the shift from glycolytic to mitochondrial pathway, induced by O_2_/O_3 _treatment, was transient and fully reversible.

Injured endothelial cells reduce mitochondrial functions [26]. Since O_2_/O_3 _treatment improves endothelial respiration, with a more elevated production of ATP, we can argue that O_2_/O_3 _could preserve endothelial cells from dysfunction. Collectively, our results are the first evidence on OA mechanism of action on cell metabolism.

## Conclusions

The preservation of endothelial metabolic activity could *in vivo *limit endothelial dysfunction provoked by the Isc or Isc/R processes. Because AO but not autohemotherapy with O_2 _alone is able to reduce endothelial dysfunction caused by Isc/R, we hypothesize that O_3_, which is endowed with anti-inflammatory properties, could limit the endotehlial activation provoked by some pro-inflammatory cytokines released during Isc/R processes. Finally, since Isc and Isc/R damages represent complications in atherosclerotic processes, we propose OA as a promising intervention in atherosclerotic patients.

## Competing interests

The authors declare that they have no competing interests.

## Authors' contributions

CF performed histological, immunohisochemical analyses and confocal microscopy on HUVEC. AF performed animal studies, O2 consumption and collected samples. DB performed isolation and culture of HUVEC, measure ATP LDH and protein levels in HUVEC. CS performed Nitrite/Nitrate determination. EF coordinated the in vitro studies. MEF coordinated the in vivo studies and wrote and edited the manuscript. All authors read and approved the final manuscript.

## Pre-publication history

The pre-publication history for this paper can be accessed here:

http://www.biomedcentral.com/1471-2369/12/61/prepub
